# Inter-fractional variations in the dosimetric parameters of accelerated partial breast irradiation using a strut-adjusted volume implant

**DOI:** 10.1093/jrr/rrz061

**Published:** 2019-10-28

**Authors:** Kotaro Iijima, Hiroyuki Okamoto, Kana Takahashi, Ako Aikawa, Akihisa Wakita, Satoshi Nakamura, Shie Nishioka, Ken Harada, Ryoichi Notake, Akimoto Sugawara, Ryoichi Yoshimura, Etsuo Kunieda, Jun Itami

**Affiliations:** 1 Department of Medical Physics, National Cancer Center Hospital, Chuo-ku, Tsukiji 5-1-1, Tokyo, 104-0045, Japan; 2 Department of Radiation Oncology, National Cancer Center Hospital, Chuo-ku, Tsukiji 5-1-1, Tokyo, 104-0045, Japan; 3 Department of Radiation Oncology, Tokai University, School of Medicine, Isehara Shimokasuya 143, Kanagawa, 259-1193, Japan; 4 Department of Radiation Therapeutics and Oncology, Tokyo Medical and Dental University, Bunkyo-ku, Ushima 1-5-45, Tokyo, 113-8510, Japan

**Keywords:** inter-fraction, inter-fractional variations in dosimetric parameters, breast cancer, brachytherapy, strut-adjusted volume implant, SAVI, APBI

## Abstract

The aim of the study was to evaluate inter-fractional dosimetric variations for high-dose rate breast brachytherapy using a strut-adjusted volume implant (SAVI). For the nine patients included, dosimetric constraints for treatment were as follows: for the planning target volume for evaluation (PTV_Eval), the volume receiving 90, 150 and 200% of the prescribed dose (*V*_90%,150%,200%_) should be >90%, ≤50 cm^3^ and ≤20 cm^3^, respectively; the dose covering 1 cm^3^ (*D*_1cc_) of the organs at risk should be ≤110% of the prescribed dose; and the air volume should be ≤10% of PTV_Eval. Differences in *V*_90%,150%,200%_, *D*_1cc_ and air volume ($\Delta V$ and $\Delta D$) as inter-fractional dosimetric variations and SAVI displacements were measured with pretreatment and planning computed tomography (CT) images. Inter-fractional dosimetric variations were analyzed for correlations with the SAVI displacements. The patients were divided into two groups based on the distance of the SAVI from the surface skin to assess the relationship between the insertion position of the SAVI and dosimetric parameters. The median Δ*V*_90%,150%,200%_ for the PTV_Eval in all patients was −0.3%, 0.2 cm^3^ and 0.2 cm^3^, respectively. The median (range) Δ*D*_1cc_ for the chest wall and surface skin was −0.8% (−18.9 to 9.4%) and 0.3% (−7.6 to 5.3%), respectively. SAVI displacement did not correlate with inter-fractional dosimetric variations. In conclusion, the dose constraints were satisfied in most cases. However, there were inter-fractional dosimetric changes due to SAVI displacement.

## INTRODUCTION

Postoperative external-beam radiation therapy (EBRT) to the whole breast with 42.56 Gy in 16 fractions is currently the standard of care after lumpectomy for early breast cancer. However, it is conducted within 4–5 weeks and thus places a significant burden on the patient [1–7]. Accelerated partial breast irradiation (APBI) exclusively targets the tumor bed by using EBRT or brachytherapy. Because of its limited irradiated volume, APBI can be completed in a shorter period of time, even less than 1 week [8–14]. Brachytherapy for APBI can be performed via multiple strategies: for example, interstitial irradiation uses a multi-catheter technique and intracavitary irradiation delivered via the MammoSite® system (Hologic Inc., Bedford, MA, USA), the Contura® system (Hologic Inc., Bedford, MA, USA) or the SAVI® system (Cianna Medical, Aliso Viejo, CA, USA) [11–13].

In the National Cancer Center Hospital (NCCH) in Tokyo, Japan, APBI is performed with a high-dose rate (HDR, ^192^Ir) and a strut-adjusted volume implant (SAVI) (UMIN ID: UMIN000021237). The SAVI can be easily placed into the lumpectomy cavity. By manipulating the source dwell points and times in multiple catheters, optimized dose distribution is achieved while minimizing the dose to organs at risk (OARs). The SAVI is composed of a center catheter and 6–10 surrounding catheters (struts) and has four standard sizes: 6–1 mini, 6–1, 8–1 and 10–1 [15]. The chosen size is based on the volume of the lumpectomy cavity [15, 16]. Once inserted, the SAVI is expanded to bring the multiple struts into close contact with the lumpectomy wall; it remains in the lumpectomy cavity during the treatment sessions. APBI is performed using an ^192^Ir remote afterloading system (RALS, MicroSelectron HDR™, Nucletron, Stockholm, Sweden) with a total dose of 34 Gy in 10 fractions, twice daily for 5 consecutive days.

However, because the breast is easily deformed, the SAVI may be displaced, resulting in inter-fractional dosimetric variations. Altman *et al*. [17] analyzed shape changes in the SAVI, but only from the day of implant to the first day of treatment. To date, there are no reports on the impact of inter-fractional variations due to anatomical changes during the entire treatment.

Thus, this study aimed to evaluate the relationship between SAVI displacement and inter-fractional variations in dosimetric parameters in HDR breast brachytherapy.

## MATERIALS AND METHODS

### Patient characteristics

Breast cancer patients who underwent APBI with a SAVI at the NCCH (seven patients) and Tokyo Medical and Dental University (TMDU) (three patients) between August 2015 and February 2017 were retrospectively analyzed for inter-fractional dosimetric variations. The median age was 64 years (range, 44–84 years).

The treated regions of the breast were divided into cranial-medial (A), caudal-medial (B), cranial-lateral (C) and caudal-lateral (D) quadrants. If the treated region included two quadrants, for example, C and D, it was denoted as CD. Two patients were right C, left C and left D, respectively, and one patient was left CD, right B, right CD and right CA, respectively.

Planning computed tomography (CT) (CT_plan_) images and pretreatment CT (CT_treat_) images were used for analysis of inter-fractional variations. In seven patients, 10–11 CT images were acquired before every treatment fraction was delivered, while 6 CT images (once a day) were acquired in each of three patients. The total number of CT images obtained was 82. All treatments were used based on an initial treatment plan without modifications of the plan.

### Treatment procedure

**Fig. 1:**
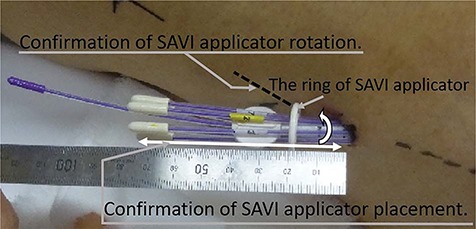
Recording of SAVI applicator length and rotation.

A SAVI of suitable size was inserted into the lumpectomy cavity and the surrounding catheters were expanded to place them directly in contact with the lumpectomy cavity wall. A CT simulation (NCCH: Aquilion™ LB, Canon Medical Systems Corporation, Tokyo, Japan; TMDU: LightSpeed Xtra, GE Healthcare, Milwaukee, Wisconsin, USA) for brachytherapy planning was performed with both of the patient’s arms up over the head for taking the exhale image. To record the SAVI position, we measured the length and rotation of the SAVI protruding from the surface skin with a ruler and drew markings on the surface skin and the ring of the SAVI, as shown in Fig. 1 (arrows).

**Fig. 2:**
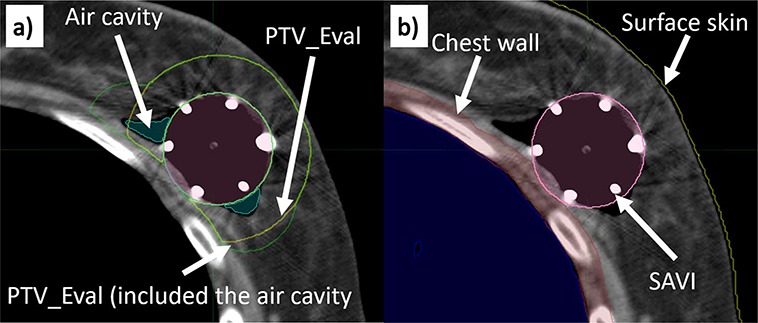
Structures used in this study. The brown, yellow, white, pink, green and orange structures are the chest wall, the surface skin, the air cavity, SAVI applicator, PTV_Eval and PTV_Eval that includes the air cavity, respectively.

CT images were transferred to a treatment planning system (Oncentra® Brachy version 4.5.1, Nucletron, Electa AB, Stockholm, Sweden). A radiation oncologist and a medical physicist delineated the expanded catheters of the SAVI (SAVI_cav_), the planning target volume (PTV), the chest wall, the surface skin and the air cavity around the SAVI. The PTV for optimization (PTV_Opt) was based on the SAVI_cav_ volume with an isotropic 1-cm expansion. The PTV for evaluation (PTV_Eval) was calculated by subtracting the volume of the SAVI_cav_, chest wall and surface skin with 2-mm thickness from the PTV_Opt (Fig. 2) [18–20].

After these structures were delineated, a treatment plan that allows coverage of 90% of the PTV_Eval was generated; the total dose was 34 Gy in 10 fractions over 5 days (prescribed dose: 3.4 Gy per fraction for the initial CT only, twice daily at intervals of least 6 h). The dose was calculated using a TG-43-based algorithm, and the grid resolution of the dose calculation was 1 mm.

To detect displacement of the SAVI, CT images were acquired before each fraction was delivered or once daily. The inter-fractional dosimetric variations of the PTV_Eval and the volumes of the chest wall, surface skin and air cavity adjacent to the SAVI were assessed on every CT image obtained before every treatment fraction was delivered.

This retrospective study was approved by the Institutional Ethical Review Board of the national cancer center (approval number: 2014–039) and was performed in accordance with the ethical standards of the 1964 Declaration of Helsinki and its later amendments.

### Inter-fractional variations in SAVI position

**Fig. 3:**
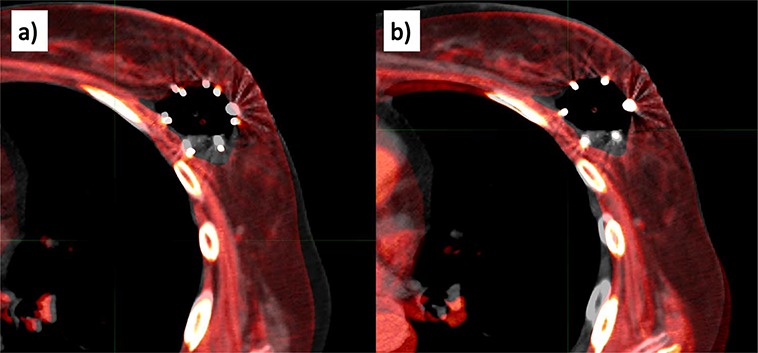
Difference in registration methods. (**a**) The registration image in which CT_treat_ (blue line) was aligned to CT_plan_ (yellow line) based on bony anatomical structure, and (**b**) the registration image in which CT_treat_ (blue line) was aligned to CT_plan_ (yellow line) using a point-based registration of five markers of SAVI applicator.

**Fig. 4:**
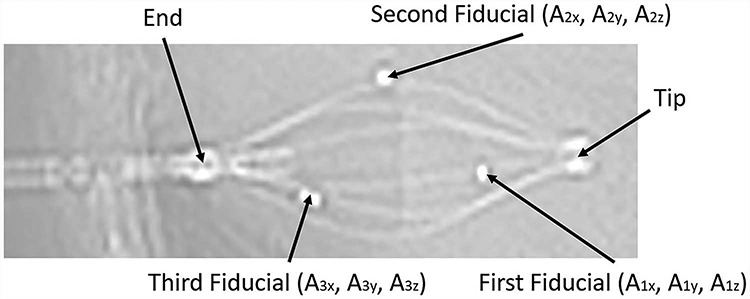
Scout CT image of SAVI.

To match the positional coordinates (Fig. 3a) and assess SAVI displacement, CT_treat_ images were aligned to CT_plan_ images based on bony anatomical structures using MIM Maestro® version 6.7.6 software (MIM Software Inc., Cleveland, OH, USA). As shown in Fig. 4, the SAVI has three fiducial markers. After the bony anatomical match, a coordinate (*A*_ix_, *A*_iy_, *A*_iz_) of the *i*th fiducial marker was manually determined by visual inspection according to a CT coordinate. *x* is the medial–lateral direction, *y* is the cranial–caudal direction, *z* is the anterior–posterior direction. The centroid (G*_x_*, G*_y_*, G*_z_*) was then calculated as:

$${\mathrm{G}}_{x,y,z}=\left(\sum_{i=1}^3\frac{A_{ix}}{3},\kern0.5em \sum_{i=1}^3\frac{A_{iy}}{3},\kern0.5em \sum_{i=1}^3\frac{A_{iz}}{3}\right).$$

The three-dimensional vector of SAVI displacement in each treatment fraction was calculated as

$${\Delta \mathrm{S}}_{x,y,z}={\mathrm{G}}_{x,y,z}^{treat}-{\mathrm{G}}_{x,y,z}^{plan},$$

where ΔS*_x,y,z_* is the 3D vector of SAVI displacement, ${\mathrm{G}}_{x,y,z}^{treat}$ is the centroid of the CT_treat_ image, and ${\mathrm{G}}_{x,y,z}^{plan}$ is the centroid of the CT_plan_ image. To eliminate interobserver variations, the fiducial marker position was determined by the same medical physicist.

### Inter-fractional variations in dosimetric parameters

A different registration technique was applied to our analysis. In Oncentra Brachy, all CT_treat_ images are aligned to CT_plan_ images using point-based registration for the five points of the SAVI (Fig. 3b): three fiducials on the surrounding catheters, the tip of the SAVI and the end of the SAVI (Fig. 4). In our study, the registration accuracies of the five points were within ±1 mm in all patients, indicating that SAVI deformation did not occur during treatment. All structures delineated on each CT_treat_ image were copied to the CT_plan_ image after registration.

The dosimetric parameters were the volume of the PTV_Eval receiving the prescribed dose, the dose to the OARs (*D*_1cc_) and the air volume. For the PTV_Eval, the volume receiving 90 (*V*_90%_), 150 (*V*_150%_) and 200% (*V*_200%_) of the prescribed dose should be >90%, ≤50 cm^3^ and ≤20 cm^3^, respectively [21–24]. The dose covering 1 cm^3^ (*D*_1cc_) of the OARs (defined here as the surface skin and chest wall) should be <110% of the prescribed dose (Table 1). The air volume should be ≤10% of the PTV_Eval. According to the National Surgical Adjuvant Breast and Bowel Project (NSABP) B-39/RTOG 0413 protocol, the air volume should be included in the PTV_Eval when it exceeds 10% of the PTV_Eval. Thus, assessing changes in air volume is essential during treatment sessions [21, 22]. The dosimetric parameters were determined for all treatment fractions to learn whether the constraints were satisfied.

**Table 1:** Dosimetric criteria for treatment planning

Dosimetric index		Planning criteria
PTV_Eval	*V*_90%_ [%]	Volume receiving 90% of the prescrived dose >90%
	*V*_150%_ [cm^3^]	Volume receiving 150% of the prescrived dose ≤50 cm^3^
	*V*_200%_ [cm^3^]	Volume receiving 200% of the prescrived dose ≤20 cm^3^
Patient’s skin	*D*_1cc_ [%]	1 cm^3^ volume receiving dose <110%
Chest wall	*D*_1cc_ [%]	1 cm^3^ volume receiving dose <110%

PTV_Eval = evaluated planning target volume.

### Correlation analysis between SAVI displacement and the dosimetric parameters

The correlation between the 3D vector of SAVI displacement (ΔS*_x,y,z_*) and the inter-fractional dosimetric variations was determined. SAVI displacement was assessed as described above.

For the PTV_Eval, inter-fractional variations in *V*_90%_, *V*_150%_ and *V*_200%_ with reference to the CT_plan_ image were calculated as:

$$\Delta{V}_{90\%,150\%,200\%}\ \left[\%\mathrm{or}\ {cm}^3\right]={V}_{90\%,150\%,200\%}^{\mathrm{treat}}-{V}_{90\%,150\%,200\%}^{\mathrm{plan}},$$

where ${V}_{90\%,150\%,200\%}^{\mathrm{treat}}$ and ${V}_{90\%,150\%,200\%}^{\mathrm{plan}}$ are *V*_90%_, *V*_150%_ and *V*_200%_ from the CT_treat_ and CT_plan_ images, respectively.

For the surface skin and chest wall, inter-fractional variations in *D*_1cc_ with reference to the CT_plan_ image were calculated as:

$${\Delta D}_{1\mathrm{cc}}\ \left[\%\right]={D}_{1\mathrm{cc}}^{\mathrm{treat}}-{D}_{1\mathrm{cc}}^{\mathrm{plan}},$$

where ${D}_{1\mathrm{cc}}^{\mathrm{treat}}$ and ${D}_{1\mathrm{cc}}^{\mathrm{plan}}$ are the *D*_1cc_ from the CT_treat_ and CT_plan_ images, respectively. The difference in doses was normalized to the prescribed dose (100%).

The Kendall rank test was used as the nonparametric correlation coefficient test, as determined using the Shapiro-Wilk normality test.

Analysis of the relationship between the dosimetric parameters and the insertion position of the SAVI

The relationship between inter-fractional variations in the insertion position of the SAVI and the inter-fractional variations in dosimetric parameters was determined. The insertion position of the SAVI was defined as the shortest length between the OARs and the edge of the SAVI on the CT image. The shortest length was chosen as it is potentially associated with changes in the skin dose. It was measured on all CT images for all patients. Nine patients were divided into two groups based on the difference in the shortest length between the CT_plan_ and CT_treat_ images: >1 cm (group A) and ≤1 cm (group B). The Wilcoxon signed-rank test was used as the nonparametric correlation coefficient test, as determined via the Shapiro-Wilk normality test.

## RESULTS

Patient no. 4 was excluded from the analysis owing to unstable placement of the SAVI during the treatment, which required correction at each session by the physician.

### Inter-fractional variations in SAVI position

**Fig. 5:**
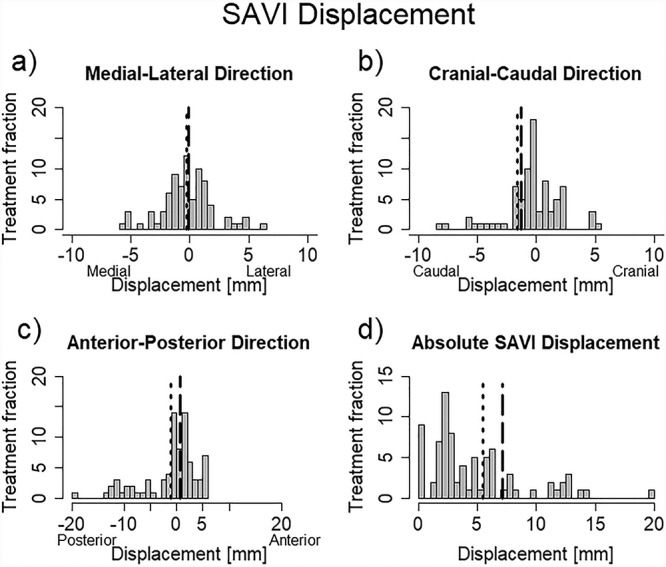
Histogram of the 3D vector of SAVI applicator displacement. (a), (b), (c) and (d) Displacement of medial–lateral, cranial–caudal, anterior–posterior directions and the absolute three-dimensional displacement, respectively. The dashed line is the mean displacement in group A. The dotted line is the mean of displacement in group B.

Figure 5 illustrates the three-dimensional vector of SAVI displacement. The mean of median (range) absolute SAVI displacement in all patients was 3.0 (2.2–12.5) mm. The maximum displacement was ~20 mm. The corresponding values for displacement in the medial–lateral (ΔS*_x_*), cranial–caudal (ΔS_y_) and anterior–posterior (ΔS_z_) directions in all patients and all fractions were 0.0 (−3.2 to 1.6), −1.2 (−4.5 to 1.4) and −4.9 (−9.7 to 3.2) mm, respectively. The rate of SAVI displacement ≥10 mm was 19.3%, and the rate of ΔS_x,y,z_ displacement ≥5 to ≤−5 mm was 5.7, 5.7 and 26.4%, respectively. These findings show that the SAVI easily moves in the anterior–posterior direction.

### Inter-fractional variations in the dosimetric parameters

**Fig. 6:**
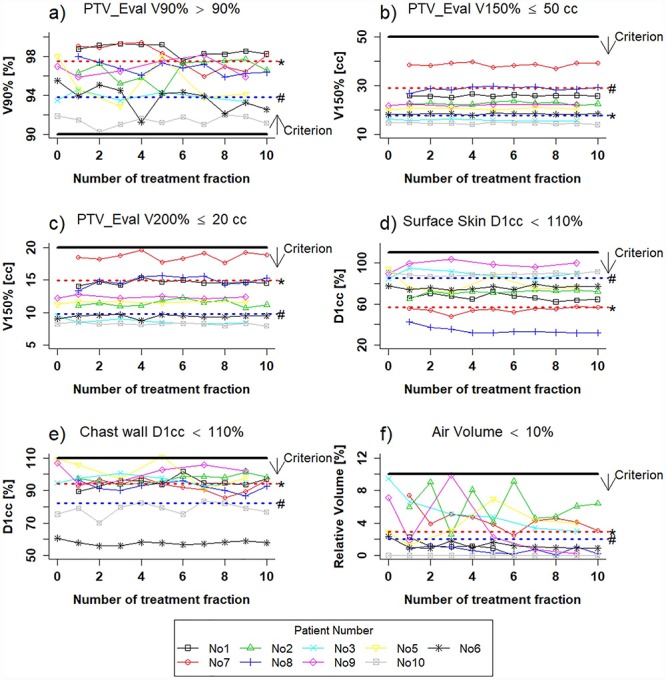
Plot diagram of the criteria and inter-fractional dosimetric parameters in all patients and all fractions. (a), (b) and (c) *V*_90%_, *V*_150%_ and *V*_200%_, respectively. (d) and (e) The *D*_1cc_ of surface skin and chest wall, respectively. (f) The relative volume of the air cavity with reference to PTV_Eval. The red (^*^) and blue (#) dotted line are the mean of the dosimetric parameters in group A and group B.

The relative volume (mean ± 1 standard deviation) of the air cavity with reference to the PTV_Eval was 2.6 ± 1.3%; the air cavity volume did not exceed 10% of the PTV_Eval. The criteria for the PTV_Eval (*V*_90%_ > 90%, *V*_150%_ ≤ 50 cm^3^ and *V*_200%_ ≤ 20 cm^3^) and OARs (*D*_1cc_ to the surface skin and chest wall <110% of the prescribed dose) were satisfied in 99% of all fractions. Figure 6 shows the air cavity volume, the *V*_90%,150%,200%_ of the PTV_Eval, and the *D*_1cc_ to the OARs in each treatment fraction; the thresholds are indicated by a solid line with an arrow.

The mean of median (range) *V*_90%,150%,200%_ of the PTV_Eval was −0.8 (−3.5 to 0.6)%, 0.4 (−0.5 to 1.5) cm^3^ and 0.5 (−0.1 to 1.8) cm^3^, respectively. The mean of median *D*_1cc_ to the surface skin and chest wall was −1.3% (−18.9 to 9.4%) and −0.9% (−7.6 to 3.6%), respectively. Although the *D*_1cc_ to the OARs varied, the PTV_Eval coverage had no large variations.

### Correlation between SAVI displacement and the dosimetric parameters

**Fig. 7:**
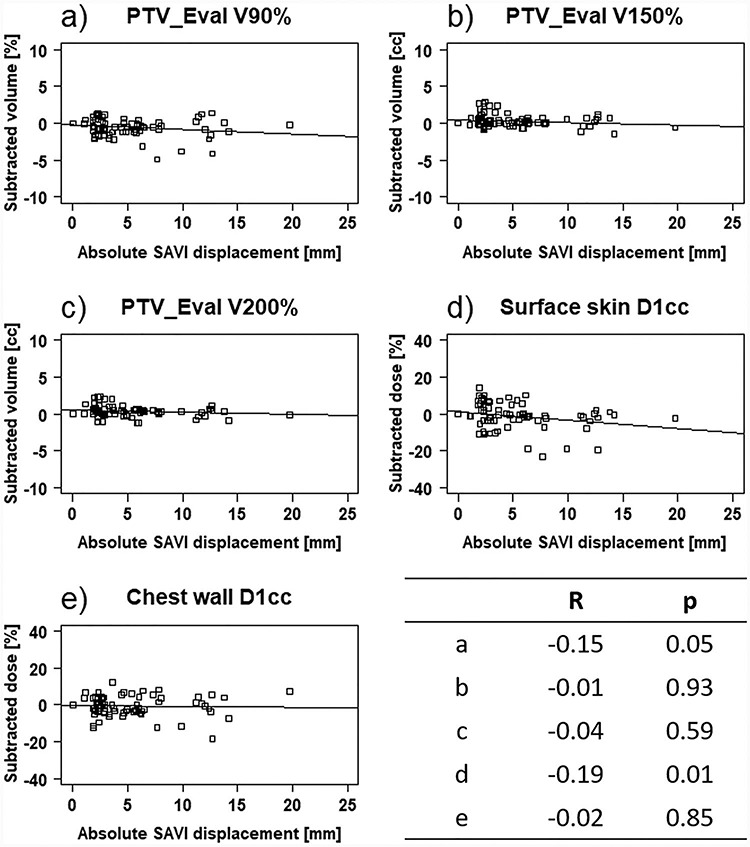
Correlation diagrams between the inter-fractional variations in dosimetric parameters and the absolute 3D vector of SAVI applicator displacement. (a), (b) and (c) The correlation diagram between ${\Delta V}_{90\%,150\%,200\%}$ of PTV_Eval and the absolute $\Delta \mathrm{S}$ of SAVI applicator. (d) and (e) The correlation diagram between ${\Delta D}_{1 cc}$ the surface skin, the chest wall and the absolute $\Delta \mathrm{S}$ of SAVI applicator.

Figure 7 shows the correlation between absolute SAVI displacement and the inter-fractional variations in dosimetric parameters, along with the correlation coefficient *R* and *P* value. There was no correlation between the Δ*V*_90%,150%,200%_ for the PTV_Eval and SAVI displacement (*R* = −0.15, −0.01 and − 0.04, and *P* = 0.05, 0.93 and 0.59, respectively). For the OARs, the Δ*D*_1cc_ for the chest wall did not correlate with SAVI displacement (*R* = −0.02, *P* = 0.85), while the Δ*D*_1cc_ for the surface skin slightly but significantly correlated with SAVI displacement (*R* = −0.19, *P* = 0.04). Therefore, no association existed between absolute SAVI displacement and the inter-fractional variations in dosimetric parameters, except the surface skin.

### Relationship between the insertion position of the SAVI and the dosimetric parameters

The median (range) length of the shortest distance between the surface skin and SAVI in group A (four cases) and B (five cases) was 16.0 (9.3–30.8) and 2.4 (2.0–6.0) mm, respectively.

In group A, the Δ*V*_90%,150%,200%_ (mean ± 1SD) for the PTV_Eval was −0.4 ± 1.1%, 0.7 ± 1.1 cm^3^ and 0.7 ± 0.8 cm^3^, respectively. In group B, the corresponding values were −0.9 ± 1.4%, 0.1 ± 0.4 cm^3^ and 0.0 ± 0.5 cm^3^, respectively. The mean Δ*D*_1cc_ for the surface skin and chest wall was −0.8 ± 6.4% and 0.3 ± 4.9% in group A and −1.3 ± 8.5% and −1.9 ± 6.1% in group B, respectively. We compared 1SD in the PTV_Eval with the OARs and confirmed that changes in the OARs were greater than those in the PTV_Eval (i.e. the inter-fractional variations in the position of the SAVI influenced the OARs more than the PTV_Eval). The Δ*V*_150%,200%_ for the PTV_Eval differed significantly between the groups (*P* < 0.05 for both), whereas the Δ*V*_90%_ and Δ*D*_1cc_ did not. The mean intergroup difference in the *V*_150%,200%_ was ~1 cm^3^, which is very small.

## DISCUSSION

3D vectors of SAVI displacement and the inter-fractional variations in dosimetric parameters were analyzed using CT_plan_ and CT_treat_ images. All treatment fractions for all patients were analyzed. In total, we found 19.3% of fractions with absolute SAVI displacement ≥10 mm, and this displacement occurred in the anterior–posterior direction (Table 2). Although the maximum SAVI displacement was ~20 mm, the dosimetric constraints were satisfied for all treatment fractions and in all patients, except patient no. 8 (Fig. 6).

**Table 2:** The median (range) difference in dosimetric parameters at all treatment fractions and all patients. Group A is a case in which SAVI is distant from the surface skin and group B is a case in which SAVI is close to the surface skin

		Median (range)						
		PTV_Eval			Surface skin		Chest wall	
Groups	Case number	*ΔV*_90%_ [%]	*ΔV*_150%_ [cm^3^]	*ΔV*_200%_ [cm^3^]	*ΔD*_1 cc_ [%]	*ΔD*_1 cc_ [cGy]	*ΔD*_1 cc_ [%]	*ΔD*_1 cc_ [cGy]
Group A	No. 1	−0.1 (−1.2 to 0.5)	0.0 (−0.8 to 0.7)	0.6 (0.0 to 1.3)	0.0 (−3.6 to 5.8)	0.0 (−12.2 to 19.7)	5.3 (0.0 to 12.2)	18.0 (0.0 to 41.5)
	No. 2	0.0 (−2.2 to 1.4)	0.1 (−1.5 to 1.2)	0.1 (−0.9 to 1.1)	−0.8 (−8.1 to 1.9)	−2.7 (−27.5 to 3.7)	0.3 (−7.3 to 5.5)	1.0 (−24.8 to 18.7)
	No. 3	0.6 (−2.1 to 1.4)	0.4 (−0.4 to 1.4)	0.2 (−0.4 to 0.2)	7.1 (0.0 to 8.6)	24.1 (0.0 to 29.2)	0.3 (−3.5 to 4.0)	1.0 (−11.9 to 13.6)
	No. 5	−1.2 (−2.1 to 0.0)	2.1 (0.0 to 2.9)	1.8 (0.0 to 2.4)	−10.1 (−11.1 to 0.0)	−34.3 (−37.7 to 0.0)	−3.9 (−9.7 to 0.0)	−13.3 (−33.0 to 0.0)
Group B	No. 6	0.2 (−0.1 to 1.0)	−0.5 (−0.7 to 0.1)	−1.1 (−1.2 to 0.0)	4.3 (0.0 to 10.1)	14.6 (0.0 to 34.3)	1.5 (−1.8 to 6.0)	5.1 (−6.1 to 20.4)
	No. 7	−0.3 (−1.1 to 1.1)	1.5 (−1.4 to 3.5)	0.1 (−0.1 to 0.6)	9.4 (0.0 to 14.2)	32.0 (0.0 to 48.3)	−4.5 (−12.7 to 0.0)	−15.3 (−43.2 to 0.0)
	No. 8	−3.5 (−4.9 to 0.1)	0.6 (0.0 to 0.9)	0.3 (0.0 to 0.5)	−18.9 (−23.4 to 0.0)	−64.3 (−79.6 to 0.0)	−7.6 (−18.7 to 1.5)	−25.8 (−63.6 to 5.1)
	No. 9	−0.4 (−1.6 to 0.1)	−0.1 (−0.7 to 0.2)	0.0 (−0.3 to 0.3)	−2.0 (−3.8 to 1.0)	−6.8 (−12.9 to 3.4)	3.6 (−5.3 to 8.2)	12.2 (−18.0 to 27.9)
	No. 10	−1.0 (−2.3 to 0.0)	0.2 (−0.4 to 0.5)	0.5 (−0.3 to 0.6)	−1.3 (−3.8 to 2.0)	−4.4 (−12.9 to 6.8)	−3.0 (−4.8 to 0.0)	−10.2 (−16.3 to 0.0)
Median of all patients		−0.3 (−3.5 to 0.6)	0.2 (−0.5 to 2.1)	0.2 (−1.1 to 1.8)	−0.8 (−18.9 to 9.4)	−2.7 (−64.3 to 32.0)	0.3 (−7.6 to 5.3)	1.0 (−25.8 to 18.0)

PTV_Eval = evaluated planning target volume.

Only the fifth *D*_1cc_ to the chest wall in patient no. 8 exceeded the constraint: the median of maximum Δ*D*_1cc_ was 5.3% and the planned *D*_1cc_ was 108%. Similar situations may also occur in other OARs such as the surface skin. For example, the maximum Δ*D*_1cc_ was 12.2% for the chest wall and 14.2% for the surface skin in all patients.

The air volumes were within 10% of the PTV_Eval in all patients and all fractions. Bhatt *et al*. [25] assessed the influence of the seroma volume surrounding a Contura® applicator on dosimetric changes in the PTV_Eval. They found that the emerging seroma decreased the *V*_90%_ of the PTV_Eval by only ~1%, even at a volume of 10 cm^3^. In our study, the maximum median air volume in all patients was 3.7 cm^3^. Hence, inter-fractional variations in air volume may not affect dosimetric parameters.

As shown in Fig. 7, there was no association between the Δ*V*_90%,50%,200%_ for the PTV_Eval and absolute SAVI displacement. When patient no. 8 was included in the analysis, the Δ*D*_1cc_ for the surface skin weakly correlated with SAVI displacement (*R* = −0.19, *P* = 0.01); however, there was no correlation (*R* = −0.12, *P* = 0.13) when she was excluded. Hence, in most cases, the inter-fractional variations in dosimetric parameters were unrelated to SAVI displacement.

**Fig. 8:**
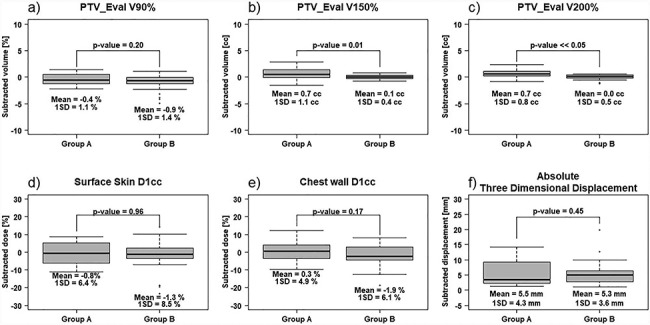
Boxplot diagrams of inter-fractional variations in dosimetric parameters between both groups. (a), (b) and (c) The PTV_Eval ${\Delta V}_{90\%,150\%,200\%}.$ (d) and (e) The surface skin and chest wall ${\Delta D}_{1 cc}$, respectively. (f) The absolute 3D displacement of SAVI applicator in both groups.

The insertion position of the SAVI strongly affected the Δ*V*_150%,200%_ for the PTV_Eval (Fig. 8). However, the influence of Δ*V*_150%,200%_ on the PTV_Eval was small: the difference in values between groups A and B was <1 cm^3^ and the influence on PTV_Eval was small. There was no difference in the Δ*D*_1cc_ for the OARs (surface skin and chest wall) between the groups. This finding indicates that the Δ*D*_1cc_ is unaffected by the insertion position of the SAVI, which was measured from the surface skin.

In our study, the measured dosimetric values for the PTV_Eval and OARs satisfied our criteria in 99% of all fractions. This confirms that neither SAVI displacement nor SAVI positioning affected the inter-fractional variations in dosimetric parameters. The *D*_1cc_ to the OARs of small-breasted patients tended to be higher than large-breasted patients, and satisfying the constraints was more difficult in the former because the SAVI is inevitably close to the OARs. Because even minimal displacement alters the *D*_1cc_ to the OARs (Fig. 8), physicians should ensure that the dosimetric constraints for the OARs are met during the treatment sessions when the patient has small breasts and the *D*_1cc_ to the OARs in the initial plan is high. Therefore, the dosimetric parameters such as *D*_1cc_ in the treatment plan should be much lower than the dose constraints. By determining the effects of the inter-fractional variations, we have shown the possibility that APBI with a SAVI can be more safely and precisely performed.

Altman *et al*. observed the displacement of SAVI when they compared the CT after insertion of the SAVI and the CT of the treatment day [17]; however, they did not visually confirm the rotation and drop-out of the SAVI as we did. Nevertheless, the number of replans was small when using the 6–1 and 6–1 mini. Vadim *et al*. concluded that their “recommendation is to carefully assess apparent rotation of the device before each treatment by repeating CT of the patient or by taking 2D radiographic images” [26]; they did not mention rotation due to the difference in size of SAVI. From their results, it is considered that the displacement of SAVI is larger when the size of SAVI is larger than 6–1. In our study, 8 cases out of 9 used the 6–1 or 6–1 mini. From the results of this previous research and ours, it is considered that the change in dosimetric parameters due to the displacement of SAVI may be small when using the 6–1 and 6–1 mini. In our protocol, CT images for confirmation were frequently acquired before the delivery of each treatment fraction, resulting in high radiation exposure, which is undesirable in some cases. Our findings show that such CT scans are not needed in cases where the SAVI used the 6–1 and 6–1 mini, visual confirmation is carried out, the SAVI is several millimeters away from the OARs and the dosimetric parameters are considerably lower than the dose constraints.

Our study had several limitations. First, it had a small number of patients and was retrospective, therefore, multivariable analysis was not performed. Because multifactorial analysis is insufficient, there may be other relevant factors in the results of this study. Second, the registration accuracies of the five points were within ±1 mm for all patients and SAVI deformation was not observed during the treatment sessions. Therefore, the inter-fractional variations in dosimetric parameters were influenced only by the movement and deformation of the breasts. The absence of SAVI deformation may reflect our use of smaller SAVIs (all patients were sizes 6–1 and 6–1 mini except one patient, who was size 8–1). Altman *et al.* [17] have shown that SAVI deformation more easily occurs with large applicators than small applicators. Third, to confirm the position of the SAVI, exhale CT images were acquired before delivery of every treatment fraction; however, the exhale phases did not remain the same in every CT scan. Hence, exhale-induced variations may have contributed to SAVI displacement. Fourth, we evaluated only changes of dose constraints, and we could not investigate changes of dose distribution, i.e. other dose levels were not examined. Despite these limitations, this study is important because it investigated the influence of SAVI displacement on dosimetric parameters in detail. This study provides helpful information for preparing robust treatment plans that consider inter-fractional SAVI displacement.

In conclusion, treatment plans usually do not consider inter-fractional variations in dosimetric parameters. However, our study shows inter-fractional changes in dosimetric parameters due to SAVI displacement. Thus, to certainly satisfy dose constraints, the dosimetric parameter of the initial plan should be much lower than that specified in dose constraints. In particular, physicians should consider administering irradiation treatment at levels below normal dose constraints by approximately 10% for the *D*_1cc_ of OARs and approximately 3% for the *V*_90%,150%,200%_ of PTV_Eval.
